# Characterization of Ceftazidime Resistance Mechanisms in Clinical Isolates of *Burkholderia pseudomallei* from Australia

**DOI:** 10.1371/journal.pone.0030789

**Published:** 2012-02-21

**Authors:** Derek S. Sarovich, Erin P. Price, Alex T. Von Schulze, James M. Cook, Mark Mayo, Lindsey M. Watson, Leisha Richardson, Meagan L. Seymour, Apichai Tuanyok, David M. Engelthaler, Talima Pearson, Sharon J. Peacock, Bart J. Currie, Paul Keim, David M. Wagner

**Affiliations:** 1 Center for Microbial Genetics and Genomics, Northern Arizona University, Flagstaff, Arizona, United States of America; 2 Menzies School of Health Research, Darwin, Northern Territory, Australia; 3 Translational Genomics Research Institute, Flagstaff, Arizona, United States of America; 4 Department of Microbiology and Immunology, Faculty of Tropical Medicine, Mahidol University, Bangkok, Thailand; 5 Department of Medicine, University of Cambridge, Cambridge, United Kingdom; University of Birmingham, United Kingdom

## Abstract

*Burkholderia pseudomallei* is a Gram-negative bacterium that causes the serious human disease, melioidosis. There is no vaccine against melioidosis and it can be fatal if not treated with a specific antibiotic regimen, which typically includes the third-generation cephalosporin, ceftazidime (CAZ). There have been several resistance mechanisms described for *B. pseudomallei*, of which the best described are amino acid changes that alter substrate specificity in the highly conserved class A β-lactamase, PenA. In the current study, we sequenced *penA* from isolates sequentially derived from two melioidosis patients with wild-type (1.5 µg/mL) and, subsequently, resistant (16 or ≥256 µg/mL) CAZ phenotypes. We identified two single-nucleotide polymorphisms (SNPs) that directly increased CAZ hydrolysis. One SNP caused an amino acid substitution (C69Y) near the active site of PenA, whereas a second novel SNP was found within the *penA* promoter region. In both instances, the CAZ resistance phenotype corresponded directly with the SNP genotype. Interestingly, these SNPs appeared after infection and under selection from CAZ chemotherapy. Through heterologous cloning and expression, and subsequent allelic exchange in the native bacterium, we confirmed the role of *penA* in generating both low-level and high-level CAZ resistance in these clinical isolates. Similar to previous studies, the amino acid substitution altered substrate specificity to other β-lactams, suggesting a potential fitness cost associated with this mutation, a finding that could be exploited to improve therapeutic outcomes in patients harboring CAZ resistant *B. pseudomallei*. Our study is the first to functionally characterize CAZ resistance in clinical isolates of *B. pseudomallei* and to provide proven and clinically relevant signatures for monitoring the development of antibiotic resistance in this important pathogen.

## Introduction

*Burkholderia pseudomallei*, the etiologic agent of melioidosis, is a saprophytic bacterium that is commonly found in surface waters and soil of Australia and Thailand. There is no effective vaccine against melioidosis, and reducing mortality from infection is based on effective antimicrobial therapy combined with supportive care. *B. pseudomallei* has a large accessory genome [1], [2] and is intrinsically resistant to many antibiotics, including gentamicin, streptomycin, rifampicin, erythromycin and many β-lactams [3], [4]. There are several different mechanisms of antibiotic resistance in *B. pseudomallei*, including multi-drug efflux pumps [5], [6], enzymatic inactivation [7], [8], impermeability of the bacterial cell membrane [9] and mutations in the antibiotic target site [10]. This impressive array of intrinsic resistance and broad-spectrum mechanisms limits the number of treatment options for melioidosis. Successful treatment of melioidosis is protracted and typically involves two stages comprising an intravenous (IV) phase followed by prolonged oral eradication therapy [11]. In Australia, the IV drug of choice for treating melioidosis is ceftazidime (CAZ), although the carbapenem drugs meropenem or imipenem are used for severe infection or in the event of treatment failure. The oral eradication phase consists of trimethoprim-sulfamethoxazole (TMP-SMX) (in combination with doxycycline in Thailand) or amoxicillin-clavulanate (AMC), and is given for up to six months because of the frequency of relapse upon termination of treatment with shorter therapy [12]. In Thailand, CAZ is the IV antibiotic of choice [11]. Thus, CAZ is the single most important antibiotic for the treatment of melioidosis.

The vast majority of *B. pseudomallei* strains are susceptible to CAZ, imipenem, meropenem, TMP-SMX, doxycycline and AMC, although a small percentage of isolates display primary resistance [4]. Of great concern to clinicians is the potential for this bacterium to develop resistance during the course of chemotherapy, especially to the first line therapy, CAZ. Although primary resistance of *B. pseudomallei* to CAZ is rare, the prolonged nature of melioidosis treatment increases the likelihood that acquired resistance can develop, especially if monotherapy is used or if the infection relapses and CAZ is employed multiple times in the same patient. Such acquired resistance has important ramifications due to the high morbidity and mortality associated with this infectious disease and the paucity of alternate treatment options.

Determining the molecular basis of CAZ resistance (CAZ^R^) ultimately provides the genetic targets needed for improved treatment outcomes for melioidosis patients by allowing clinicians to rapidly and inexpensively monitor the emergence of resistant populations. It has been previously shown that mutations in the *B. pseudomallei* class A β-lactamase (encoded by the gene, *penA*) may confer CAZ^R^ [13]–[15]. These studies identified mutations in the *penA* gene of CAZ^R^ strains that caused amino acid alterations around conserved motifs. However, functional characterization of *penA* in clinical isolates of *B. pseudomallei* has not yet been explored. Thus, there is a need to pinpoint the precise molecular mechanisms behind CAZ^R^ in clinical *B. pseudomallei* isolates that correlate with the CAZ^R^ phenotypes observed by clinicians.

In the current study, we determined the molecular mechanisms for CAZ^R^ in *B. pseudomallei* strains from two Australian melioidosis patients who temporally developed resistant CAZ^R^ strains during CAZ therapy. To confirm that CAZ^R^ developed *in vivo* and was not the result of re-infection with a resistant strain, we subjected the patient isolates to multilocus variable-number tandem repeat analysis (MLVA) and multilocus sequence typing (MLST). In addition, we screened a large panel of clinical and environmental *B. pseudomallei* for CAZ^R^ mechanisms using allele-specific real-time PCR to determine the rate of primary CAZ^R^ in this bacterium. Last, we tested a panel of β-lactams to determine the suitability of these alternate antibiotics for treating CAZ^R^ *B. pseudomallei* clinical isolates.

## Materials and Methods

### Ethics statement

Ethics approval was granted by the Human Research Ethics Committee of the Northern Territory Department of Health and the Menzies School of Health Research (HREC 04/09), with written informed consent obtained from patients.

### *B. pseudomallei* clinical isolates used in this study

#### Melioidosis “Patient 21”

Three isolates from Patient 21 (P21) [16], [17] were used for this study (Table 1). The first two isolates were susceptible to CAZ (CAZ^S^), whereas the most recent isolate, MSHR 99, displayed CAZ^R^ (16 µg/mL; Table 1). P21, a 63 y.o. male with Type 2 diabetes, chronic renal disease and hazardous alcohol use from Darwin, Australia, was diagnosed with melioidosis following *B. pseudomallei* isolation from blood cultures (isolate MSHR 73). The patient was treated with IV CAZ and TMP-SMX for two weeks and was discharged on doxycycline. The patient had recrudescence of disease three months later, and *B. pseudomallei* was again isolated from blood cultures (MSHR 95). Following further treatment with CAZ the patient was placed on oral AMC, but subsequently deteriorated (MSHR 99 from blood culture) and died 4 months after his initial admission.

**Table 1:** *Burkholderia pseudomallei* isolates obtained from two relapsed melioidosis patients, Patient 21 and Patient 337, and their corresponding ceftazidime MICs.

Isolate ID^a^	Patient	Date of isolation	Site of isolation	CAZ^b^ MIC (µg/mL)	*penA* sequence
MSHR 73	21	11-Jan-91	Blood	1.5	w.t.
MSHR 95	21	3-Apr-91	Blood	1.5	w.t.
MSHR 99	21	3-May-91	Blood	16	*penA* -21A
MSHR 1141	337	13-Mar-01	Sputum	1.5	w.t.
MSHR 1225	337	25-Jul-01	Throat	1.5	w.t.
MSHR 1226	337	10-Aug-01	Throat	≥256	*penA* 281A
MSHR 1298	337	09-Oct-01	Throat	≥256	*penA* -21A, 281A
MSHR 1300	337	09-Oct-01	Rectal swab	≥256	*penA* -21A, 281A
MSHR 1302	337	09-Oct-01	Unknown	≥256	*penA* -21A, 281A

a MSHR, Menzies School of Health Research;

b CAZ, ceftazidime.

#### Melioidosis “Patient 337”

Six *B. pseudomallei* isolates derived from an individual (P337) suffering from relapsing melioidosis were obtained for this study. Although the earliest two isolates obtained from this patient were CAZ^S^, four latter isolates harbored a high-level CAZ^R^ phenotype (≥256 µg/mL; Table 1). P337, a 61 y.o. male with Type 2 diabetes and metastatic bronchogenic carcinoma, likely contracted melioidosis following environmental exposure with *B. pseudomallei-*contaminated soil. Upon initial admission and *B. pseudomallei* isolation (isolate MSHR 1141), P337 was placed on IV CAZ for four weeks, followed by oral TMP-SMX and doxycycline. In addition to antimicrobial therapy, the patient required immunosuppressive therapy for their malignancy. A four-month follow-up revealed that P337 was still culture-positive for *B. pseudomallei* (MSHR 1225) despite being on oral doxycycline. Intravenous CAZ was re-administered for three weeks followed by maintenance therapy comprising oral doxycycline and chloramphenicol. P337 remained culture positive for *B. pseudomallei* and strains isolated between five to seven months after the initial diagnosis displayed CAZ^R^ (MSHR 1226 onwards). P337 remained on oral antibiotics but succumbed to the bronchogenic carcinoma shortly thereafter.

### Bacterial growth conditions

*B. pseudomallei* isolates were grown on Luria-Bertani agar (LBA) (Beckton-Dickinson, Franklin Lakes, NJ) at 37°C for 24 h to 48 h. All *Escherichia coli* strains (Supplemental Table S1) including “*Escherichia cloni*” 10G (Lucigen, Middleton, WI) were grown on LBA at 37°C for 24 h. Antibiotic susceptibility testing was performed on Mueller-Hinton agar (Hardy Diagnostics, Santa Maria, CA) at 37°C.

### Antibiotic sensitivity testing

A previous study has shown that B-lactam MIC testing in *B. pseudomallei* is independent of salt concentrations [15]. Therefore, MICs were determined solely using E-tests (bioMérieux, Durham, NC) according to the manufacturer's instructions.

### PCR amplification, sequencing and cloning of *penA*

PCR amplification of the *Burkholderia penA* gene was performed using HotStarTaq Master Mix (Qiagen, Valencia, CA) with the addition of betaine (Sigma Aldrich, St. Louis, MO) to a final concentration of 1.8 M, using the following primers (5′-3′); penA_F (CGCCACAAATTCGCACGCAC) and penA_R (GCGACTCGCGCTCCGTGAAC) (IDT, Coralville, IA). The thermocycling conditions were 95°C for 15 min followed by 35 cycles of 30 s at 95°C, 30 s at 65°C, and 1 min at 72°C, with a final extension of 10 min at 72°C. Cloning of *penA* PCR products was performed using the pGC™ Blue Vector (Lucigen) according to the manufacturer's instructions. Sanger sequencing was used to identify polymorphisms, to determine insert orientation and to verify presence of insert. Nucleotide sequences of *penA* from CAZ^S^ and CAZ^R^ *B. pseudomallei* isolates from P21 and P337 have been submitted to NCBI.

### Allelic exchange of *penA*

Allelic exchange was performed using the suicide vector pMo130 [18], which is compliant for use with Select Agent bacteria such as *B. pseudomallei*. We used this allelic exchange system to remove *penA* from both CAZ^S^ and CAZ^R^ isolates, enabling us to determine whether this single gene was responsible for the CAZ^R^. Briefly, both the upstream and downstream regions of *penA* were amplified using the following respective primers (5′-3′); penA_US_R (AAGCGGTCAGATCTTCCGCGTTGTGCTGGA) and penA_US_F (GCATATCTGCTAGCTCTGTTGCGGCATCGCTTT), penA_DS_R (CCGAGATCTTCACGGAGCGCGAGTC) and penA_DS_F (GACAAGCTTGAAAAACAGGGCGAACGCACAGG). These primers amplify approximately 800–1000 bp regions flanking *penA* but do not amplify the gene itself. Underlined nucleotides represent deliberately introduced restriction enzyme (RE) sites, which are required for ligation into pMo130. Amplification was performed using slowdown PCR as previously described [19] without the addition of the altered dGTP analog. Following PCR, amplicons were purified using a PCR purification kit (Qiagen). The upstream product was digested with *Nhe*I (Promega, Madison, WI) and *Bgl*II (Promega), and the downstream fragment was digested with *Bgl*II and *Hin*dIII (Promega) according to the manufacturer's instructions. It is important to note that target amplicons did not contain these RE sites to ensure intact, full length amplicons upon digestion. Following digestion, products were ligated into *Nhe*I and *Hin*dIII digested pMo130 to create pMo130-US-DS. Correct orientation and incorporation of PCR products in the final construct were verified by multiple RE analyses. The remaining allelic exchange procedure was performed as previously described [18].

### *Cis* complementation of *penA*

The *penA* gene was reintroduced into Δ*penA* strains according to previously described methods [18] that are in compliance with Select Agent Rule 42 CFR Part 73 (http://www.selectagents.gov/Regulations.html). First, the upstream region was amplified using the primers described previously. Following amplification, the upstream region was digested with *Nhe*I and *Bgl*II and was introduced into pMo130 digested with *Nhe*I and *Bgl*II to create pMo130-US. Second, *penA* was amplified from MSHRs 663 (*penA* ^+^), 99 (*penA* minus(−)21A; 16 µg/uL CAZ^R^), 1226 (*penA* 281A; ≥256 µg/uL CAZ^R^) or 1300 (*penA* -21A, 281A; ≥256 µg/uL CAZ^R^) depending on the desired construct (Supplemental Table S1). Amplification of *penA* was performed using the following primers (5′-3′); penA_comp_F (GTTCAGCAGATCTAACAGATCGCCGAGATGG) and penA_comp_R (GCACCGCGATATCTCGCGCTCCGTGAACCTT) with underlined nucleotides representing deliberately introduced *Bgl*II and *Eco*RV restriction sites, respectively. Third, the downstream (DS) region of *penA* was amplified using the following primers; penA_DScompF (5′-CTTCCGGATATCTCACGGAGCGCGAGTC) and penA_DScompR (5′-CGACGACAAGCTTGAAAAACAGGGCGAACGCACAGG). Underlined nucleotides represent *Eco*RV and *Hind*III sites, respectively. Last, the amplified fragment containing *penA* was digested with *Bgl*II and *Eco*RV and the DS fragment was digested with *Eco*RV and *Hin*dIII, and both DS and *penA* fragments were ligated into pMo130-US digested with *Bgl*II and *Hin*dIII. The final construct was verified by restriction analyses (data not shown). Importantly, the *penA* amplicon included 200 bp of the *penA* upstream region, such that promoter sequences were incorporated into the pMo130-US-*penA-*DS vectors to ensure expression when re-introduced into *B. pseudomallei*. Following creation, the pMo130-US-*penA-*DS construct was introduced into the *B. pseudomallei* Δ*penA* strains and the gene deletion was reversed following previously published protocols [18].

### *penA* SNP characterization using PCR

Genomic DNA obtained from approximately 2,400 genetically and geographically diverse *Burkholderia* spp. isolates (Price EP et al., manuscript in preparation) was used to screen for the presence of the mutant *penA* 281A SNP in these isolates. These samples largely comprise clinical and environmental *Burkholderia* isolates from Australia and Thailand, but also other isolates collected from around the world. DNA was extracted using the DNeasy tissue extraction kit (Qiagen), 5% chelex-100 heat soak [20] or the Wizard genomic DNA purification kit (Promega). DNA was normalized to 1 ng/µL using the NanoDrop 8000 spectrophotometer (Thermo Scientific, Waltham, MA) prior to PCR analysis.

We developed a SYBR Green-based mismatch amplification mutation assay (SYBR MAMA) protocol for interrogating the mutant and wild-type variants at the *penA* -21A and *penA* 281A SNPs in *B. pseudomallei* and *Burkholderia mallei*. Due to the highly conserved nature of *penA* among near-neighbor *Burkholderia* spp., these assays also produce amplicons for *B. oklahomensis*, *B. thailandensis*, *B. vietnamiensis*, *B. humptydooensis* sp. nov. and *B. ubonensis*, albeit at lowered efficiency due to primer-template mismatches. SYBR MAMA exploits the differential amplification efficiency of allele-specific primers for SNP interrogation [21], [22]. In real-time PCR, this differential efficiency is observed by measuring the cycles to threshold (C_T_) of both allele-specific primers to determine the nucleotide present at the SNP. For *penA* -21A interrogation, two allele-specific primers, 21promA_99_F (5′-CACTCCTGTGACGAGAGCTGATTCA) and 21promG_wt_F (5′-CACTCCTGTGACGAGAGCTGATTCG) and one common reverse primer 21prom_comR (5′-GGCGACGTTTTTCGCTTGG) were used to interrogate the SNP. For *penA* 281 primers, penA_281-G (5′-GGCGACGAGCGTTTCCCGTTATG) and penA_281-A (5′-TTTTTTTTTTTTTTTCGACGAGCGTTTCCCGTTATA) were used in combination with penA_281_R (5′-CGCAGCGCAAAGCATCAT) to interrogate the SNP. PenA_281-A amplifies the mutant allele, which confers an enhanced ability to degrade CAZ, whereas *penA* ^+^ is preferentially amplified by penA_281-G. PCRs consisted of one allele specific primer and the appropriate reverse primer per well. All samples were run in duplicate. A total 0.3 uM of each primer (IDT), 1× SYBR Green PCR Master Mix (Applied Biosystems, Foster City, CA), and molecular grade water (GIBCO, Carlsbad, CA) were added to a volume of 9 µL. One µL of DNA template was added to the reaction. All PCRs were conducted using an ABI PRISM 7900HT real-time PCR instrument (Applied Biosystems) and default cycling conditions that comprised 2 min at 50°C, 10 min at 95°C, followed by 40 cycles of 15 sec at 95°C and 1 min at 60°C. A dissociation curve was performed following amplification to confirm amplicon specificity. MSHR 1300 was used as a positive control for the mutant alleles.

### 16S rDNA PCR

To verify bacterial DNA quality, we ran a control 16S PCR against all DNA samples using previously published primers [23]. All 16S PCRs were performed on the ABI PRISM 7900HT real-time PCR instrument (Applied Biosystems) using default cycling conditions.

### Multiple locus variable number tandem repeat analysis (MLVA)

MLVA was performed on the P21 and P337 isolates as previously described [24], with the exclusion of locus 20 k, to rule out re-infection with different strains.

### Multi-locus sequence typing (MLST)

MLST was performed as described elsewhere [25].

## Results

### Sequencing of *penA* from P21 and P337 isolates

We sequenced *penA* of *B. pseudomallei* isolates obtained from two melioidosis patients (P21 and P337; Table 1; Genbank accession numbers JQ364927 through JQ364935) where CAZ^R^ appeared to have developed directly in response to CAZ chemotherapy. All isolates from P21 (MSHRs 73, 95 and 99) demonstrated the same PenA amino acid composition and were identical to the wild-type PenA of CAZ^S^ *B. pseudomallei* K96243. However, analysis of the promoter region uncovered a novel G to A nucleotide transition (referred to herein as *penA* -21A) in the latter isolate, MSHR 99, which was not present in either MSHRs 73 or 94 (Table 1) (Note that mutation numbering was determined from the whole genome annotation of *B. pseudomallei* 1106a due to the absence of *penA* signal peptide annotation in *B. pseudomallei* K96243). Importantly, the G to A transition corresponded to a 10-fold increase in CAZ^R^ (16 µg/mL), suggesting this SNP is involved in up-regulation of *penA* expression.

The majority of CAZ^R^ isolates from P337 also showed the same putative regulatory mutation in the promoter region (MSHRs 1298, 1300 and 1302). In addition, we identified a mutation in *penA* at position 281 in most of the latter isolates that resulted in a cysteine to tyrosine (C69Y) substitution adjacent to the ^70^SXXK^73^ conserved motif (Ambler's numbering scheme) [26]. This SNP has been previously identified in CAZ^R^ *B. pseudomallei* isolates [14], and functionally characterized in a Select-Agent exempt strain of *B. pseudomallei* [15]. The mutated *penA* (referred to herein as *penA* 281A) directly corresponded to high-level CAZ^R^ (≥256 µg/mL; Table 1), resulting in a >170-fold increase in CAZ hydrolysis, similar to a previous report [15]. Isolates MSHRs 1141 and 1225 contained *penA* ^+^, whereas isolates subsequently collected from P337 (Table 1), with the exception of MSHR 1226, contained both *penA* -21A and *penA* 281A mutations. The dual-mutant *penA* isolates from P337 also yielded CAZ MICs of ≥256 µg/mL. Interestingly, MSHR 1226, an isolate collected mid-infection, contained only the *penA* 281A mutation and not the promoter region - 21A SNP.

### Comparison of native and heterologous expression of *penA* variants

We used a heterologous cloning and expression approach to better understand the link between *penA* mutations, CAZ^R^ and substrate specificity and compared these data with *penA* behavior in the native host, as a previous study has demonstrated that heterologous hosts can be useful for approximating the activity of *B. pseudomallei* PenA towards β-lactam substrates [13]. We used an *E. cloni* system to express *penA* amplified from MSHRs 99 (-21A mutant), 1226 (281A mutant) and 1300 (-21A and 281A dual mutant) and a CAZ^S^ *penA*+ control strain (MSHR 663). MICs of the *E. cloni* host were compared to *E. cloni* expressing the *penA* variants to determine background CAZ hydrolysis (Table 2).

**Table 2:** MICs of *B. pseudomallei* isolates with and without *penA* and heterologously expressed genes in “*E. cloni*”.

Bacterial strain	β-lactam^a^^,^ b								
	CAZ	AMP	AMX	AMC	CRO	CEC	CLA	CT-CTL	MEPM
*E. cloni* pGC - no insert	0.25	2	8	2	0.13	2	0.19	<0.25/0.064	0.023
*E. cloni* pGC-*penA* ^+^	0.25–0.5	12	24	4	1.5	6	0.19	<0.25/0.094	0.023
*E. cloni* pGC-*penA* -21A	0.25–0.5	16	36	5	1.5	6	0.19	<0.25/0.125	0.023
*E. cloni* pGC-*penA* 281A	3–4	2	8	4	0.5	4	0.19	0.25/0.125	ND
*E. cloni* pGC-*penA* -21A, 281A (dual mutant)	8	2	8	2	0.75	2	0.19	<0.25/0.125	0.023
*Burkholderia pseudomallei*									
MSHR 73 (*penA* ^+^)	1.5	24	≥256	1.5	16	≥256	1.5	>16/>1	1
MSHR 95 (*penA* ^+^)	1.5	24	≥256	1.5	12	≥256	1.5	>16/>1	1
MSHR 99 (*penA* -21A)	16	≥256	≥256	8	≥256	≥256	>4	>16/>1	1.5
MSHR 99 Δ*penA*	1	2	3	1.5	1.5	48	1	2/>1	ND
MSHR 99 *penA* -21A (*cis* complement)	16	≥256	≥256	12	≥256	≥256	>4	>16/>1	ND
MSHR 99 *penA* ^+^ (*cis* complement)	2	16	192–256	2	16	≥256	1.5	>16/>1	ND
MSHR 1225 (*penA* ^+^)	1	8	≥256	1.5	8	≥256	0.75	>16/>1	ND
MSHR 1298 (*penA* -21A, 281A) (dual mutant)	≥256	8	32	1.5	≥256	≥256	4	>16/>1	ND
MSHR 1302 (*penA* -21A, 281A) (dual mutant)	≥256	8	32	1.5	≥256	≥256	4	>16/>1	ND
MSHR 1141 (*penA* ^+^)	1.5	24	≥256	1.5	8	≥256	0.75	>16/>1	ND
MSHR 1141 Δ*penA*	1	2	4	1.5	1.5	24	0.75	>16/>1	ND
MSHR 1141 *penA* ^+^ (*cis* complement)	1.5	24	≥256	1.5	8	≥256	0.75	>16/>1	ND
MSHR 1226 (*penA* 281A)	≥256	3	12	2	96	≥256	3	>16/>1	ND
MSHR 1226 Δ*penA*	1	1.5	4	1.5	1	16	0.75	1/>1	ND
MSHR 1226 *penA* 281A (cis complement)	≥256	ND	ND	ND	ND	ND	ND	ND	ND
MSHR 1226 *penA* ^+^ (*cis* complement)	2	24	≥256	2	16	≥256	3	>16/>1	ND
MSHR 1300 (*penA* -21A, 281A)	≥256	8	32	1.5	≥256	≥256	3	>16/>1	4
MSHR 1300 Δ*penA*	0.5	1.5	3	1	0.75	16	0.75	1.5/>1	4
MSHR 1300 *penA* ^+^ (*cis* complement)	2	12	192–256	1.5	8	≥256	0.75	>16/>1	ND
MSHR 1300 *penA* -21A, 281A (*cis* complement)	≥256	8	24	1.5	≥256	≥256	2–3	>16/>1	ND

a CAZ, ceftazidime; AMX, Amoxicillin; AMP, Ampicillin; AMC, Amoxicillin-clavulanic acid; CRO, ceftriaxone; CEC, cefaclor; CLA, ceftazidime-clavulanic acid; CT-CTL, cefotaxime/cefotaxime-clavulanic acid; MEPM, meropenem; ND, not determined.

b MICs presented in µg/mL.

Neither the heterologously expressed PenA^+^ or the -21A *penA* mutant altered the CAZ MIC in *E. cloni* despite a 16-fold increase in CAZ MIC in *B. pseudomallei* -21A *penA*, indicating lowered sensitivity of the heterologous system. MSHR 1226 *penA* (containing the 281A mutation) increased degradation of CAZ by 8-fold and MSHR 1300 (dual *penA* mutant) by 16-fold, which mimicked the increases observed in the native *B. pseudomallei* host, albeit with substantially lower fold differences. Interestingly, increased activity towards CAZ by both the dual and C69Y-mutated PenA mutants was accompanied by a decrease in hydrolytic activity for amoxicillin (AMX), and to a lesser extent ampicillin (AMP) (Table 2). In contrast, we saw an increase in MICs in *penA -*21A against a range of β-lactams. This phenomenon was most evident when comparing AMX MICs, where *E. cloni* expressing *penA* -21A yielded a 1.5-fold increase compared with *penA* ^+^. In *B. pseudomallei*, both *penA -*21A and *penA^+^* gave AMX MICs of ≥256 µg/mL, indicating that higher MIC detection would be required to confirm these heterologous AMX MIC differences in the native host. These results demonstrate that the amino acid mutation in PenA is highly favorable for CAZ hydrolysis but causes a substantial reduction in hydrolytic activity towards at least two other β-lactams, as previously shown [15]. The *penA* -21A mutant mutation also enhances hydrolysis of CAZ, although not to the level of the C69Y mutant. However, *penA* -21A causes upregulation of *penA*, which enhances hydrolysis towards other β-lactam substrates, including antibiotics containing clavulanic acid.

### Knockout and complementation of *penA*

Following confirmation of CAZ^R^ by heterologous expression of *penA*, this gene was removed from mutant and w.t. *B. pseudomallei* strains (CAZ^R^ MSHR 99, CAZ^S^ MSHR 1141 and CAZ^R^ MSHRs 1226 and 1300) to determine CAZ MICs in the original host compared with its *penA* knockout. Following *penA* removal, we screened for CAZ MICs in *penA* knockouts. All Δ*penA* strains possessed a CAZ^S^ phenotype, with MICs of approximately 1 µg/mL (Table 2). To further verify the role of *penA* in CAZ^R^, we complemented all strains with *penA* ^+^ to test for restoration of wild-type CAZ^S^ and AMX^R^ phenotypes. Finally, we reinserted the original *penA* genes back into CAZ^R^ strains to examine reproducibility of CAZ^R^ phenotypes (Table 2). When *penA* ^+^ was introduced into the MSHR 99, MSHR 1226 or MSHR 1300 Δ*penA* strains, the wild-type resistance profile was restored indicating that these mutations were the sole cause of CAZ^R^ in these strains. Further evidence of a single-gene phenotype was confirmed following restoration of the CAZ^R^ MICs in CAZ^R^ strains complemented with their native *penA* (Table 2).

### Prevalence of *penA* mutants in *Burkholderia* spp

Once it was established that *penA* 281A and *penA* -21A were responsible for conferring CAZ^R^ in the *B. pseudomallei* strains from our study, we determined the frequency of these mutations across clinical and environmental *Burkholderia* isolates. We obtained the most robust allelic discrimination using the SYBR MAMA format (Figure S1). Following verification of assay performance, the *penA* SYBR MAMA assays were screened across approximately 2400 *Burkholderia* spp. isolates derived from clinical and environmental sources. Screening for these mutations across our *Burkholderia* DNA collection revealed that only MSHR 1226, 1298, 1300 and 1302, all isolates from P337, contained the mutant *penA* 281A allele. In contrast, we identified two additional isolates carrying the *penA* -21A mutation. One isolate was from a clinical case in Malaysia and the second was an environmental isolate from undisturbed soil in Northeast Thailand.

### Clonality of P21 and P337 infection

We were interested in determining if the isolates obtained from P21 and P337 were clonal, suggestive of a relapsed infection and *in vivo* development of CAZ^R^ rather than re-infection with a different CAZ^R^ strain. To determine the clonality of infection, we carried out 22-locus MLVA [24] on the nine isolates from the two patients. MLVA targets rapidly mutable loci throughout the genome; therefore, unrelated isolates are highly likely to display distinct MLVA profiles, making this method indispensable for determining *in vivo* infection clonality [24], [27].

In P21, MLVA failed to show any variability among the three strains across all 22-loci. In P337, MLVA demonstrated 12 mutations among all six strains, ranging from a two-repeat insertion to a five-repeat deletion (data not shown). These mutation rates are within the realm of expected *in vivo* mutation rates of clonal *B. pseudomallei* isolates within a single host, as determined in previous studies [24], [27]. Further, there was no evidence of a temporal distinction between MLVA mutants, with mutations occurring across all timepoints (data not shown).

MLST was also performed on the patient isolates to consolidate our conclusion of clonality from the MLVA profiles. As expected, MLST genotypes were identical within patients, with P21 isolates being sequence type (ST) 135 and P337 isolates genotyping as ST-330. These results are unsurprising as infection relapse is more common than re-infection [28]–[30].

## Discussion

Melioidosis is a serious disease without an effective vaccine that requires prolonged antibiotic therapy to eradicate. Due to the intrinsic resistance of *B. pseudomallei* to a wide range of antibiotics, the treatment options for melioidosis are unfortunately limited to a small number of antimicrobial agents. Primary treatment usually consists of IV CAZ followed by prolonged oral antibiotic therapy with a secondary drug such as trimethoprim-sulfamethoxazole, doxycycline or AMC [11]. Although primary resistance to these clinical drugs is rare [4], development of resistance can result as a consequence of the prolonged therapy typically needed for treating melioidosis, especially in cases of recurrent melioidosis, which afflicts approximately 10% of patients [31]. Due to the heavy reliance on CAZ as first line therapy for melioidosis, both primary and secondary CAZ^R^ pose a significant challenge in treatment and play a critical role in patient outcomes.

Most cases of melioidosis are treated with IV CAZ monotherapy in the initial eradication phase, followed by a change in antimicrobial drugs once the patient starts oral therapy. The switch in treatment probably abrogates the selective pressure on CAZ^R^ mutants to arise and become dominant *in vivo*. However, clinicians may employ CAZ multiple times or for an extended length during the course of melioidosis, particularly in recurrent cases, a strategy that leads to an increased potential for CAZ^R^ to develop. In the current study, we observed *B. pseudomallei* develop both low-level and high-level CAZ^R^ in direct response to chemotherapy with CAZ in two separate cases of recurrent melioidosis. Both patients suffered relapse within months of initial infection and were treated with IV CAZ as the primary treatment in both instances. In the first patient, P21, *B. pseudomallei* evolved low-level resistance due to a SNP located -21 bp upstream of the putative *penA* start codon, which resulted in an approximate 10-fold up-regulation of the class A β-lactamase PenA. This promoter mutation caused resistance to not only the first line treatment, CAZ, but alarmingly, the follow-up AMC chemotherapy. In the second patient, P337, high-level CAZ^R^ developed due primarily to an amino acid substitution in PenA that altered the substrate specificity of this enzyme, increasing CAZ^R^ by at least 170-fold. Interestingly, the same promoter mutation altering PenA expression was also observed in many isolates from P337, suggesting continued selection pressure for increased *penA* expression. We strongly suspect that the repeated treatment with CAZ in these relapsed melioidosis patients likely provided the prolonged selective pressure needed for CAZ^R^ mutations to develop and become dominant within the *in vivo* bacterial population. Although more recurrent melioidosis cases would need to be investigated to confirm this hypothesis, our study demonstrates that there is a risk for treatment failure associated with repeated CAZ chemotherapy in relapsing melioidosis patients that is worthy of further study.

Although high-level CAZ^R^ is very uncommon in *B. pseudomallei*, it has been previously reported. Sam and colleagues [14] isolated a CAZ^R^ strain (24 µg/mL) from a patient who later harbored *B. pseudomallei* with high-level CAZ^R^ (≥256 µg/mL), indicating a potential stepwise mutation progression in CAZ^R^. In the current study, we did not detect a low-level CAZ^R^ isolate from P337 (all isolates obtained over the course of infection were either CAZ^S^ or showed high-level CAZ^R^), suggesting that the single *penA* 281A mutation (C69Y) was responsible for the high-level resistance phenotype. Alteration of this amino acid yielded a CAZ^R^ MIC of ≥256 µg/mL in a Select Agent exempt strain of *B. pseudomallei* [15]. Our study confirmed these previous results based on isolate MSHR 1226, which contained this single mutation and was resistant to CAZ at ≥256 µg/mL. However, the small sample size (*n* = 6) used in the current study renders the possibility that the low-level CAZ^R^ phenotype was missed during sampling. In addition, Sam and co-workers [14] saw an increased resistance to AMC for their initial isolates that we did not identify in P337. However, we observed a similar resistance profile towards other β-lactams in the P21 isolate, MSHR 99. It is interesting to speculate whether the initial isolates from the Sam *et al*. study possessed an alteration in the *penA* promoter region, similarly to MSHR 99, as the MIC values for both AMC and CAZ are identical between studies.

Having demonstrated both heterologously and in the native host that the 281A and -21A *penA* mutants were responsible for CAZ^R^ in these isolates, we were interested in determining the frequency of these mutations over a large collection of *B. pseudomallei* from both clinical and environmental origins. PCR screening of over 2400 samples (of which none are known to be from other recurrent melioidosis patients) showed that no other isolates with the PenA C69Y mutation were found, indicating that this mutation is fortunately rare. We propose several reasons for the low frequency of C69Y in *B. pseudomallei*. First, *B. pseudomallei* is a soil dwelling organism, yet CAZ is a synthetically manufactured antibiotic that does not naturally occur in the environment [32] and thus there is no selection pressure to develop CAZ^R^ in the environment. Second, multiple molecular mechanisms likely exist for generating CAZ^R^ in *Burkholderia* spp. [13], [14]. Third, there appears to be a trade-off for high-level resistance to CAZ in the form of increased susceptibility to the other β-lactams (Table 2), which provides a heavy selective disadvantage to *B. pseudomallei* in the face of β-lactamases produced by other soil-borne microbes. In other words, the *penA*281A mutation is only favorable to *B. pseudomallei* during an *in vivo* infection that includes CAZ as a chemotherapeutic agent.

Unlike *penA*281A, our screening efforts did identify two additional isolates with the *penA* -21A mutation, totaling approximately 0.1% prevalence within our *B. pseudomallei* collection. One of these mutants belonged to a Malaysian melioidosis case. We lack clinical data on this patient so cannot determine whether this adaptation was acquired *in vivo* and as a result of treatment with CAZ. The *penA* -21A mutation was not restricted to clinical isolates, being found in an environmental sample from an undisturbed soil location in Northeast Thailand. Unlike *penA*281A, the *penA* -21A mutation caused cross-resistance to all the β-lactams tested, including AMC, which contains a β-lactamase inhibitor. The infrequency of both *penA* mutations identified in this study supports the continued usefulness of CAZ as a first line treatment option for melioidosis. However, caution should be exercised when administering any antibiotic to melioidosis patients, particularly in light of our study. Ideally, clinicians should ascertain the MIC status of strains during the course of treatment and adjust therapy accordingly. In particular, *B. pseudomallei* strains that possess low-level CAZ^R^, such as the P21 *penA* -21A mutants, can result in treatment failure to other β-lactams, including those that contain β-lactamase inhibitors. Alternately, treatment with CAZ can supply sufficient selective pressure for *B. pseudomallei* to develop high-level CAZ^R^, as observed in P337.

The system of heterologous cloning and expression used in our study has been important in expediting the identification of genes responsible for antibiotic resistance. Due to the Select Agent classification of *B. pseudomallei*, genetic manipulation is often highly laborious and the number of genetic tools available to researchers is limited. Using DNA derived from *B. pseudomallei* and a Biosafety Level 1 heterologous screening host (such as *E. coli* K-12) has allowed us to screen larger numbers of genes for antibiotic resistance than would otherwise be possible working with the native hosts. However, a secondary method for candidate gene identification, such as allelic exchange, is still required to verify heterologous expression results as expression profiles can vary markedly between the heterologous and native bacteria. For instance, G+C content, codon usage, alterations in expression levels from non-native promoters or vector copy numbers, differences in protein folding or other post-translational modifications can result in changes of native function, leading to erroneous expression results [33], [34]. A recent study describes the approval and use of a Select Agent exempt strain of *B. pseudomallei* as a heterologous host for virulent *B. pseudomallei* counterparts [15]. This new strain will enable researchers to rapidly identify single gene candidates and allow multiple gene, random cloning approaches to be undertaken, greatly accelerating functional genomics of *B. pseudomallei*.

In conclusion, we have demonstrated that antibiotic administration in cases of chronic and recurrent bacterial infections can have a profound impact on treatment efficacy. We provide functional evidence for direct selection of *B. pseudomallei* mutants with enhanced antibiotic resistance following administration of the first line of defense, CAZ, and for one patient, both the first and second lines of defense (CAZ and AMC). Sufficient information regarding mechanisms of resistance and the development of robust PCR assays will one day allow clinicians to monitor bacterial populations in real-time, with alteration of treatment as bacterial populations are identified that develop or alter their resistance profiles. We have shown that although *B. pseudomallei* is able to develop high-level CAZ^R^ it does so infrequently and at the cost of becoming more sensitive to other antimicrobials, providing an avenue for future research in combating recurrent and chronic melioidosis. Other potential mechanisms of CAZ^R^ in *B. pseudomallei*, such as efflux pumps, alterations in other β-lactamases or changes in the cell wall, remain to be characterized. Elucidation of these resistance mechanisms will allow rapid characterization of a *B. pseudomallei* infection and appropriate treatment to be administered, reducing the morbidity and mortality of melioidosis.

## Supporting Information

Figure S1 ***B. pseudomallei*** **real-time SYBR MAMA** ***penA*** **281A SNP assay.** The left real-time PCR amplification plot demonstrates preferential amplification of *penA* ^+^ in a non-mutated *B. pseudomallei* strain (K96243), whereas the right amplification plot shows the mutant polymorphism (*penA* 281A) from *B. pseudomallei* MSHR 1300. Blue, *penA* ^+^; red, mutant *penA* 281A allele; green, no-template controls.
(TIF)

Table S1 **Non-** ***Burkholderia*** **strains and plasmids in the current study.**
(DOC)
