# Supplementary figures and images for: Characterization of Ceftazidime Resistance Mechanisms in Clinical Isolates of Burkholderia pseudomallei from Australia
Source: PLoS One. 2012 Feb 21;7(2):e30789. doi: 10.1371/journal.pone.0030789 (PMC3283585; doi:10.1371/journal.pone.0030789)

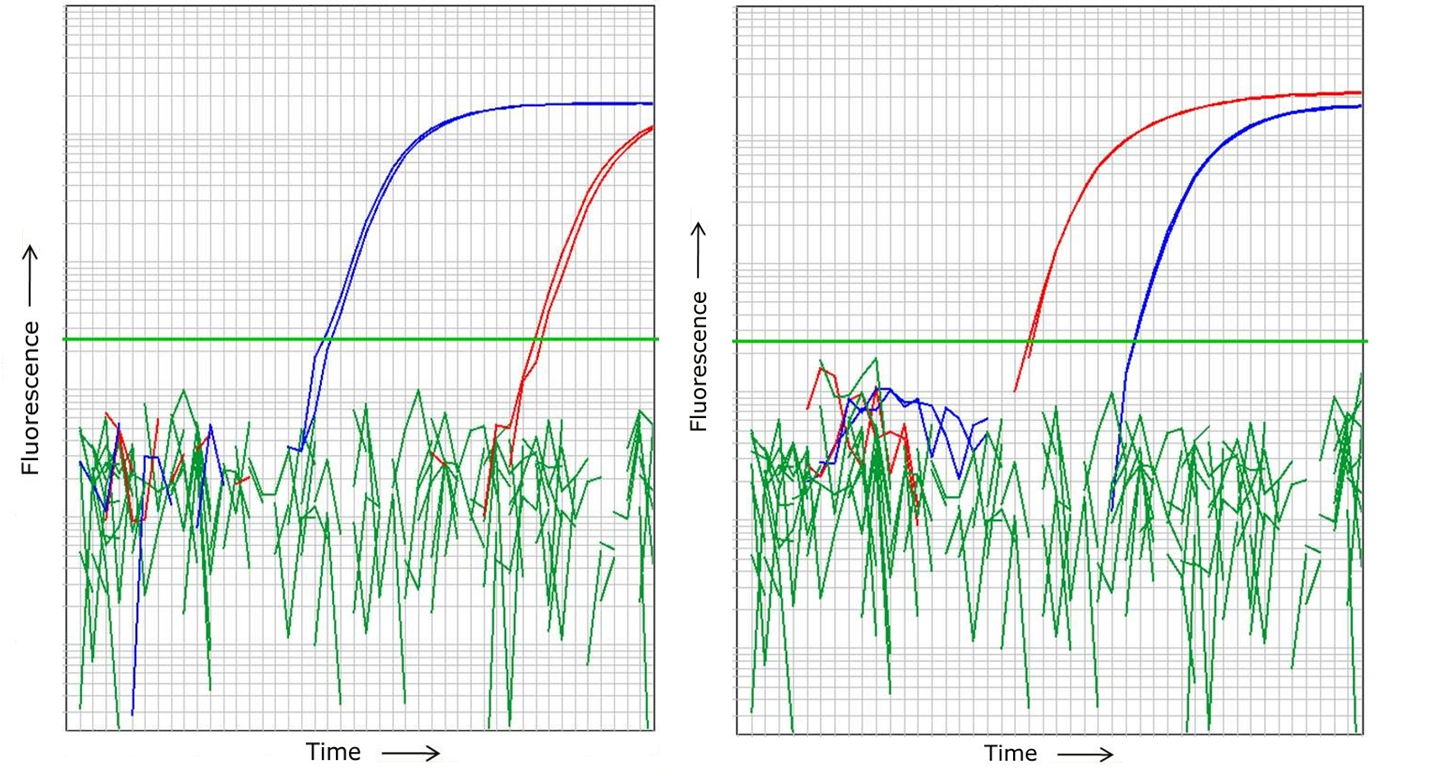

Supplement: Figure S1 — B. pseudomallei real-time SYBR MAMA penA 281A SNP assay. The left real-time PCR amplification plot demonstrates preferential amplification of penA + in a non-mutated B. pseudomallei strain (K96243), whereas the right amplification plot shows the mutant polymorphism (penA 281A) from B. pseudomallei MSHR 1300. Blue, penA +; red, mutant penA 281A allele; green, no-template controls. (TIF) [file pone.0030789.s001.tif]
