# Supplementary material for: Characterization of Ceftazidime Resistance Mechanisms in Clinical Isolates of Burkholderia pseudomallei from Australia
Source: PLoS One. 2012 Feb 21;7(2):e30789. doi: 10.1371/journal.pone.0030789 (PMC3283585; doi:10.1371/journal.pone.0030789)
Supplement: Table S1 — Non- Burkholderia strains and plasmids in the current study. (DOC) [file pone.0030789.s002.doc]

**Supplemental Table 1.** Non-*Burkholderia* strains and plasmids in the current study

| **Strain or plasmid** | **Genotype or properties** | **Source** |
| --- | --- | --- |
| *Escherichia cloni* | F¯, *mcrA,* ∆(*mrr-hsdRMS-mcrBC*), *endA*1, *recA*1, φ80d*lac*Z∆M15, ∆*lacX*74, *araD*139, ∆(*ara,leu*)7697, *galU*, *galK*, *rpsL*, *nupG*λ, *tonA* | Lucigen |
| pGC-*penA*^+^ | Cloning vector containing *pen*A from *B. pseudomallei* MSHR 663 (wild-type) | This study |
| pGC-*penA* -21A | Cloning vector containing *pen*A from *B. pseudomallei* MSHR 99 (promoter mutation) | This study |
| pGC-*penA*281A | Cloning vector containing *pen*A from *B. pseudomallei* MSHR 1226 (missense mutation) | This study |
| pGC-*penA -*21A, 281A | Cloning vector containing *pen*A from *B. pseudomallei* MSHR 1300 (promoter and missense mutation) | This study |
| pMo130 | Allelic exchange vector for Select Agent *Burkholderia* spp. |  |
| pMo130-US-DS | Allelic exchange vector containing the upstream and downstream regions of *penA* | This study |
| pMo130-US-*penA*^+^*-*DS | Same as pMo130-US-DS but with complete *penA* gene from MSHR 663 (wild-type) | This study |
| pMo130-US-*penA* -21A*-*DS | Same as pMo130-US-DS but with complete *penA* gene from MSHR 99 (promoter mutation) | This study |
| pMo130-US-*penA*281A*-*DS | Same as pMo130-US-DS but with complete *penA* gene from MSHR 1226 (missense mutation) | This study |
| pMo130-US-*penA -*21A, 281A*-*DS | Same as pMo130-US-DS but with complete *penA* gene from MSHR 1300 (promoter and missense mutation) | This study |
| *E. coli* S17-1 | *recA* *thi* *pro* *hsd*R-M^+^RP4: 2-Tc:Mu:Km Tn7 Tp^R^ Sm^R^ |  |
| *E. coli* JM109 | *recA*1, *endA*1, *gyrA*96, *thi-*1, *hsdR*17, *supE*44, *relA*1, Δ(*lac-proAB*)/F' [*traD*36, *proAB*^+^, *lacI*^q^, *lacZ*ΔM15] | Promega |
